# Neurological Dysfunction Associated with Antiphospholipid Syndrome: Histopathological Brain Findings of Thrombotic Changes in a Mouse Model

**DOI:** 10.1080/10446670410001670526

**Published:** 2004-03

**Authors:** Lea Ziporen, Sylvia Polak-Charcon, D. Amos Korczyn, Iris Goldberg, Arnon Afek, Juri Kopolovic, Joab Chapman, Yehuda Shoenfeld

**Affiliations:** 1 Research Unit of Autoimmune Diseases Department of Medicine B Sheba Medical Center Tel-Hashomer 52621 Israel; 2 Departments of Neurology and Physiology & Pharmacology Sackler School of Medicine Tel-Aviv University Tel-Aviv 69978 Israel; 3 Department of Pathology Sheba Medical Center Tel-Hashomer 52621 Israel; 4 Sieratzki Chair of Neurology Sackler School of Medicine Tel-Aviv University Tel-Aviv 69978 Israel

## Abstract

The aim of this work was to study the pathological processes underlying neurological dysfunctions displayed by BALB/C mice induced with experimental antiphospholipid syndrome (APS), as we have previously reported. Experimental APS was induced in female BALB/C mice by immunization with a pathogenic monoclonal anticardiolipin (aCL) antibody, H-3 (*n*=10), or an irrelevant immunoglobulin in controls (*n*=10). Mice immunized with H-3 developed clinical and neurological manifestations of APS, including: embryo resorption, thrombocytopenia neurological defects and behavioral disturbances. In mouse sera, the titer of various autoantibodies were elevated, including: anti-phospholipids (aPLs), anti-2 glycoprotein-I (β2GPI), anti-endothelial cell antibodies (AECA) and low titer of anti-dsDNA antibodies. Five months after APS induction, mice were sacrificed and brain tissue specimens were processed for hematoxylin and eosin (H&E), immunofluorescence staining and transmission electron microscopy (TEM). H&E staining of cortical tissue derived from all APS mice revealed mild inflammation, localized mainly in the meninges. Prominent IgG deposits in the large vessel walls and perivascular IgG leakage were observed by immunofluorescence. No large thrombi were observed in large vessels. However, EM evaluation of cerebral tissue revealed pathological changes in the microvessels. Thrombotic occlusion of capillaries in combination with mild inflammation was the main finding and may underlie the neurological defects displayed by mice with APS.


*Clinical & Developmental Immunology*, March 2004, Vol. 11 (1), pp. 67–75

Taylor & Francis healthsciences

### Neurological Dysfunction Associated with Antiphospholipid Syndrome: Histopathological Brain Findings of Thrombotic Changes in a Mouse Model

LEA ZIPOREN[a,b], SYLVIA POLAK-CHARCON[c], D AMOS KORCZYN[b,d], IRIS GOLDBERG[c], ARNON AFEK[c], JURI KOPOLOVIC[c], JOAB CHAPMAN[b] and YEHUDA SHOENFELD[a,*,†]

[a]*Research Unit of Autoimmune Diseases, Department of Medicine B, Sheba Medical Center, Tel-Hashomer 52621, Israel;* [b]*Departments of Neurology and Physiology & Pharmacology, Sackler School of Medicine, Tel-Aviv University, Tel-Aviv 69978, Israel;* [c]*Department of Pathology, Sheba Medical Center, Tel-Hashomer 52621, Israel;* [d]*Sieratzki Chair of Neurology, Sackler School of Medicine, Tel-Aviv University, Tel-Aviv 69978, Israel*

The aim of this work was to study the pathological processes underlying neurological dysfunctions displayed by BALB/C mice induced with experimental antiphospholipid syndrome (APS), as we have previously reported. Experimental APS was induced in female BALB/C mice by immunization with a pathogenic monoclonal anticardiolipin (aCL) antibody, H-3 ($n = 10$), or an irrelevant immunoglobulin in controls ($n = 10$). Mice immunized with H-3 developed clinical and neurological manifestations of APS, including: embryo resorption, thrombocytopenia neurological defects and behavioral disturbances. In mouse sera, the titer of various autoantibodies were elevated, including: anti-phospholipids (aPLs), anti-β2 glycoprotein-I (β2GPI), anti-endothelial cell antibodies (AECA) and low titer of anti-dsDNA antibodies. Five months after APS induction, mice were sacrificed and brain tissue specimens were processed for hematoxylin and eosin (H&E), immunofluorescence staining and transmission electron microscopy (TEM). H&E staining of cortical tissue derived from all APS mice revealed mild inflammation, localized mainly in the meninges. Prominent IgG deposits in the large vessel walls and perivascular IgG leakage were observed by immunofluorescence. No large thrombi were observed in large vessels. However, EM evaluation of cerebral tissue revealed pathological changes in the microvessels. Thrombotic occlusion of capillaries in combination with mild inflammation was the main finding and may underlie the neurological defects displayed by mice with APS.

*Keywords*: Antiphospholipid syndrome; Autoantibodies; Mouse; Neurological manifestations; Thrombosis

*Abbreviations*: APS, antiphospholipid syndrome; SLE, systemic lupus erythematosus; CL, cardiolipin; PLs, phospholipids; β2GPI, beta-2-glycoprotein-I; AECA, anti-endothelial cell antibodies; EC, endothelial cells; HUVEC, Human umbilical vein endothelial cells; TEM, transmission electron microscopy; H&E, hematoxylin and eosin

### INTRODUCTION

The antiphospholipid syndrome (APS) is an autoimmune disorder characterized by recurrent thrombotic (venous or arterial) events, repeated spontaneous abortions, and thrombocytopenia, in combination with persistently elevated titers of anti-phospholipid antibodies (aPL) or lupus anticoagulants (Hughes, 1993; Asherson *et al.*, 1996; Wilson *et al.*, 1999). It appears as a primary entity, or in the constellation of other autoimmune diseases, mainly systemic lupus erythematosus (SLE) (Asherson and Cervera, 1994; Asherson *et al.*, 1996). A wide spectrum of other clinical manifestations with a multi-organ involvement has been reported in association with APS (Hojnik *et al.*, 1996; Ziporen *et al.*, 1996; Levy *et al.*, 1997; Brey, 2000), including valvular heart disease, dermal, renal and neurological dysfunctions. The neurological impairments reported in association with APS (Brey, 2000) *include*: transient ischemic attacks (TIA's) and strokes in young people (Levine *et al.*, 1990; Ginsburg *et al.*, 1992; Brey *et al.*, 1993; Toubi *et al.*, 1995), amaurosis fugax (Digre *et al.*, 1989), dementia

*YS—Incumbent of the Laura Schwarz-Kipp, Chair for Research of Autoimmune Diseases, Tel-Aviv University, Israel.
†Corresponding author. Tel.: +972-3-5302652. Fax: +972-3-5352855. E-mail: shoenfel@post.tau.ac.il

ISSN 1740-2522 print/ISSN 1740-2530 online © 2004 Taylor & Francis Ltd
DOI: 10.1080/10446670410001670526

(Inzelberg *et al.*, 1992; Mosek *et al.*, 2000), seizures (Inzelberg and Korczyn, 1989; Herranz *et al.*, 1994; Verrot *et al.*, 1997) and chorea (Brey *et al.*, 1993; Toubi *et al.*, 1995). However, the underlying mechanisms responsible for the diverse neurological symptoms associated with aPL are not fully understood.

Thrombotic events are believed to underlie many of the clinical symptoms (Caudrado *et al.*, 1997), with a direct pathogenic role for thrombus formation attributed to aPL (Kornberg *et al.*, 1994; Pierangeli *et al.*, 1995). Yet, some neurological conditions, such as chorea, migraine and diffuse cerebral atrophy, are not easily explained by a solely thrombotic mechanism.

A direct interaction of aPL autoantibodies with neuronal tissue is also plausible. Indeed, a number of *in vitro* studies have shown a direct effect of aCL on neuronal function (Sun *et al.*, 1992; Liou *et al.*, 1994; Kent *et al.*, 1997; Chapman *et al.*, 1999). The aPL have been shown to interfere with astrocytes proliferation (Sun *et al.*, 1992), to reduce GABA receptor-mediated chloride currents in snail neurons (Liou *et al.*, 1994), to bind directly to neuronal tissue of different origins (Kent *et al.*, 1997) and to cause depolarization and permeabilization of synaptoneurosomes (Chapman *et al.*, 1999).

Better understanding of these questions might derive from experimental models of APS (Bakimer *et al.*, 1992; Ziporen *et al.*, 1997). Neurological involvement in experimental APS has been reported by us (Ziporen *et al.*, 1997; Katzav *et al.*, 2001; Shrot *et al.*, 2002) and by others (Kier, 1990; Smith *et al.*, 1990; Brey and Teale, 1992; Hess *et al.*, 1993; Aron *et al.*, 1995). We have demonstrated hyperactive behavior and other neurological deficits in BALB/c mice with experimental primary APS (Ziporen *et al.*, 1997). Cognitive impairments and neurological symptoms have been demonstrated also in MRL/lpr and NZB/NZW F1 lupus-prone mice. Various degrees of inflammation and thrombosis were the main pathological findings in the brains of these mice (Kier, 1990; Smith *et al.*, 1990; Brey and Teale, 1992; Hess *et al.*, 1993; Aron *et al.*, 1995). However, the specific association of similar findings with aPLs has not been demonstrated in models of primary APS.

The aim of this study was to assess the pathological changes underlying neurological and behavioral defects displayed by BALB/C mice with experimental primary APS.

### MATERIALS AND METHODS

### Animals

Female BALB/c mice were obtained from the animal house in the Tel-Aviv University Medical School. The animals were raised under standard conditions, temperature 23 ± 1°C, 12:12 h light–dark cycle with *ad-libitum* access to food and water.

### H-3 Monoclonal Antibody

H-3 is a natural human monoclonal IgM-aCL carrying a pathogenic idiotype which is common in patients with autoimmune diseases (Sutjita *et al.*, 1989) and when injected to naive BALB/c female mice induces primary APS (Bakimer *et al.*, 1992; Ziporen *et al.*, 1997). The antibody was affinity purified from the supernatant of a hybridoma cell line, kindly donated by Dr M. Sutjita, from the Flinders Medical Center, South Australia (Sutjita *et al.*, 1989). Human IgM was used as control (Jackson Immunoresearch Laboratories, West Grove, PA).

### Endothelial Cell Cultures: Human Umbilical Vein Endothelial Cells (HUVEC)

Human umbilical vein endothelial cells (HUVEC) were isolated from umbilical cord veins as described previously (Damianovich *et al.*, 1996) and used for cyto-ELISA and for FACS analysis to detect anti-endothelial cells antibodies (AECA) in the mice serum.

### Induction of Experimental APS in Mice by Active Immunization with ACL (H-3)

BALB/c mice (8–10 weeks old females, weighing 25–30 g) were immunized intradermally in the hind footpads with either 10 μg H-3 ($n = 10$), or normal human IgM ($n = 10$), emulsified in complete Freund's adjuvant (CFA, Difco Laboratories Inc., Detroit, MI). A boost injection with 10 μg of the H-3, or normal IgM in phosphate buffer saline (PBS) was administered 3 weeks later.

### Serological and Clinical Evaluation of the Mice

Mice were bled from the retro-orbital plexus at one-month intervals. Blood cell counts were conducted using a single optical cytometer (HC Plus Cell Control; Coulter Electronics) for determination of thrombocytopenia. Sera were separated and tested by ELISA for the presence of autoantibodies.

### *ELISA*

Serum samples were tested at 1:100 dilution for anticardiolipin (aCL), anti-phosphatidylserine (aPS), anti-phosphatidylinositol (aPl), anti-phosphatydilcholine (aPC), anti-$\beta_2$GPI, anti-dsDNA, anti-IgM and AECA.

Ninety six well polystyrene microtiter plates (Nunc, Roskilde, Denmark) were coated with cardiolipin (CL), phosphatidylserine (PS), phosphtidylinositol (PI), (or empty wells) at 50 μg/ml in ethanol, or with phosphatidylcholine (PC) in methanol/chloroform, then left open at 4°C until evaporation. For anti-human IgM detection, microtiter plates were coated with 2 μg/ml human IgM in 0.05 M $NaHCO_3$ buffer, pH 9.5, for overnight and washed three times with 0.05%TBS-Tween.

For the detection of anti-dsDNA, microtiter plates were coated with poly-L-lysine at 50 μg/ml in $ddH_2O$ for 30 min at RT, washed ×1 with TBS-Tween 0.1% and ×2 with TBS. Then, the wells were coated with calf thymus DNA at 2.5 μg/ml in TE for 2 h at RT, washed and neutralized with poly-L-glutamic acid at 50 μg/ml in TBS, for 1 h at 37°C.

Five percent bovine serum (BS) in tris buffer saline (TBS) was used as blocking agent, and 2% BS in TBS, as sample diluent. Washes were performed with TBS. The antibody binding was quantitated using alkaline phosphatase-conjugated goat anti-mouse IgG or IgM (Jackson Immunoresearch Laboratories, West Grove, PA), followed by *p*-nitrophenylphosphate substrate. Optical density (OD) was read at 405 nm.

For the detection of antibodies to β2 glycoprotein-I ($\beta_2$GPI), microtiter plates were coated with 2.5 μg/ml purified human $\beta_2$GPI (kindly donated by Dr A. Tincani, Brescia, Italy) in 0.05 M $NaHCO_3$, pH-9.5 for 16–18 h at 4°C, followed by three washes with PBS-0.05% Tween 20. For blocking and sample dilution, 1% BSA-PBS-0.05% Tween 20 was employed.

### *Anti-endothelial Cell Activity*

Anti-endothelial cell activity in the animal sera was measured in cyto-ELISA with unfixed HUVEC, according to a previously described protocol (Damianovich *et al.*, 1996) in microtiter plates coated with confluent HUVEC endothelial cells. Briefly, HUVEC cells were seeded in gelatin coated 96-well microtiter plates (Nunclon) at $2.5 \times 10^4$ cells/well, and were allowed to grow to confluence for 1 or 2 days. Cells were washed with Hank's balanced salt solution (HBSS) and incubated with a blocking buffer (HBSS/0.5% BSA) for 30 min at 37°C to prevent non-specific binding. After an additional wash cells were exposed to the tested sera or control antibody diluted in HBSS/10% FCS (dilution 1:25–1:200 of serum) for 60 min at room temperature. Cells were washed again and incubated with a second antibody, alkaline phosphatase-conjugated goat anti-mouse IgG (Jackson Immuno Research Laboratory) followed by *p*-nitrophenyl phosphate disodium (pNPP, Sigma, St. Louis, MO) as a substrate. The OD was read at 405 nm by ELISA plate-reader (EAR 400 AT, SLT-Labinstruments, Austria).

### Flow Cytometric (FACS) Analysis of Mouse Serum Binding to HUVEC

To detect AECA antibodies, serum pools of APS mice or controls were assessed for binding to EC by FACS. HUVEC cells ($5 \times 10^5$) in PBS-BSA 2% were incubated with the mice pooled serum in 1:25 dilution (total volume 100 ml) for 45 min in 4°C. The cells were washed twice in PBS-BSA 2% and incubated with FITC-conjugated goat anti-mouse IgG (Sigma) for 30 min in 4°C in the dark. After 3 washes with washing buffer, the cells were filtered by a nylon-mesh filter and counted in a flow cytometer (Coulter, Hialeah, FL) equipped with a 500 mW argon laser and a helium–neon laser; $10^5$ cells were tested in each experiment. The percentage of cells stained was determined.

### Histological and Immunohistochemical Evaluation of Brain Tissue Specimens

### *Perfusion and Tissue Preparation*

Mice were deeply anaesthetized with sodium pentobarbitone (Nembutal, Abbot, France), 50 mg/kg body weight, intra-peritoneal. A transcardiac perfusion was conducted with 100 ml physiologic, followed by a 10 ml mixture of 1% paraformaldehyde and 10 ml of 1.25% glutaraldehyde in PBS. A specimen from the frontal cortex of each brain was immersed in 2.5% glutaraldehyde for evaluation by electron microscopy (EM). One hemisphere of each brain was snap frozen in liquid nitrogen for immunofluorescence study, and the other hemisphere was dispersed in 4% formaldehyde buffer for hematoxylin and eosin (H&E) staining.

### Immunofluorescence Studies for the Detection of Mouse IgG Deposits

Frozen brain tissue specimens derived from 10 APS mice and 10 control mice were transversely cut into 10 μm thick sections and placed on poly-L-lysine coated slides. Sections were stained with H&E and evaluated by direct immunoflourescence, as described previously (Hojnik *et al.*, 1996). Briefly, slides were dried under airstream for 45 min, fixed in acetone for 5 min at −20°C, and rinsed three times for 5 min in BSA-TBS-Tween. FITC-conjugated primary antibody goat anti-mouse IgG (Sigma), or FITC-conjugated normal goat IgG as control, diluted in TBS, were applied for 30 min at room temperature (RT). The slides were rinsed three times for 5 min in BSA-TBS-Tween, stained with Evans blue, transferred to 95% ethanol for 2 min, dried and mounted with Entellan (Merck, Darmstadt, Germany), and examined under a fluorescence microscope (Olympus Vanox AH3).

### Transmission Electron Microscopy (TEM)

Brain samples from APS and control mice were processed for TEM. Specimens ($2 \times 2\,mm^3$) of brain tissue were fixed in 2.5% glutaraldehyde in 0.1 M cacodylate buffer (pH 7.4) for 1 h at RT. After several washings with the same buffer, the tissues were post-fixed with 2% $OsO_4$ in cacodylate buffer for 2 h, washed, and dehydrated in a graded ethanol solutions (30, 50, 75, 96, 100%) at RT. The specimens were transferred to propylene oxide and subsequently embedded in Epon embedding mixture (Bio-Rad) at 55–65°C for 3 days in a flat mold. Thin sections of 60 mμ were obtained with a Reichert ultra microtom, contrasted with uranyl acetate, followed by staining with lead citrate and examined with Joel-JEM 1200 EX II.

### RESULTS

### Serological Markers and *In Vivo* Assessment of Mice

Mice were evaluated for clinical and serological manifestations of APS (Ziporen *et al.*, 1997). As previously published (Bakimer *et al.*, 1992; Ziporen *et al.*, 1997), all the mice that had been immunized with H-3 antibody demonstrated elevated titers of circulating aPLs, including anti-CL, anti-PS and anti-PE antibodies, anti-$\beta_2$GPI antibodies, AECA, and anti-dsDNA, measured by specific ELISA assays (Bakimer *et al.*, 1992; Ziporen *et al.*, 1997). Control mice developed only anti-human IgM (hIgM). Results of antibody levels at the time point the mice were sacrificed, 5 months post immunization with aCL, are summarized in Table I. Other manifestations of APS were also found. Thrombocytopenia was detected in the APS mice (Bakimer *et al.*, 1992; Ziporen *et al.*, 1997). Number of platelets (average no. ± SD) was $653 \pm 179 \times 10^3/\mu$ in APS mice, compared to $1076 \pm 217 \times 10^3/\mu l$ in the control mice, $p < 0.05$. Fetuses resorption were measured, as previously described (Bakimer *et al.*, 1992; Ziporen *et al.*, 1997), 12/32 resorptions were detected in APS mice, compared to 5/30 in the controls, $p < 0.05$. Behavioral and neurological tests were conducted on all the mice, demonstrating behavioral changes in the APS mice including hyperactive behavior, defected performance of neurological reflexes and motor incoordination, as previously described (Ziporen *et al.*, 1997).

### Flow Cytometric Analysis Findings

To assess specific binding activity to endothelial cells (EC), in addition to ELISA test, we performed flow cytometric analysis with HUVEC cells. Pooled serum from 10 APS mice was found to bind 89% of HUVEC cells tested (Fig. 1A), compared to 27% binding activity of pooled sera from 10 control mice, as shown in Fig. 1B. These results indicate that a specific binding reactivity to

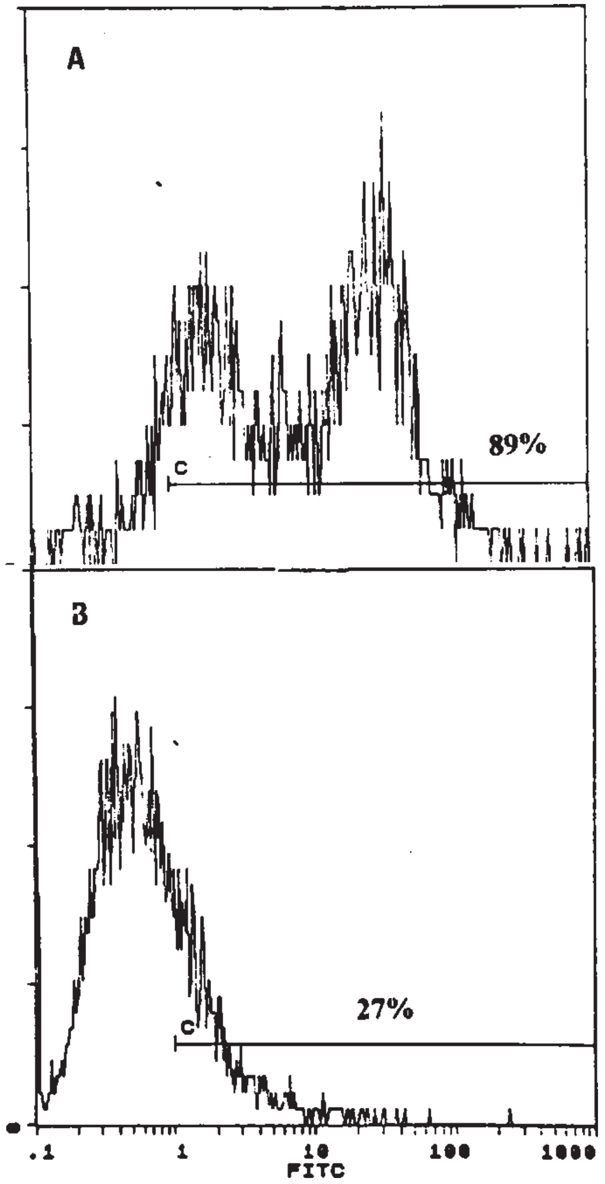

FIGURE 1 Cytofluorimetric evaluation of HUVEC endothelial cells binding activity in APS mice (A) and control (B) serum (1:25 dilution of pooled sera). Fluorescence intensity (FITC) is displayed on the horizontal axis and relative cell number on the vertical axis. (C) horizontal lines indicate fluorescent-positive cells stained with FITC-conjugated anti-mouse IgG. Prominent positive binding activity of HUVEC cells (89%) was found in serum of APS mice immunized with H-3 aCL antibody (a), control mice serum (b) show limited binding activity of HUVEC cells (27%).

TABLE I Antibodies level (average ± SD) in the serum of APS mice and controls, as tested in ELISA (1:100 dilution), 5 months after the induction of APS

| | Autoantibodies level in mouse sera (O.D.405) | | | | | | | |
|---|---|---|---|---|---|---|---|---|
| Mouse group | CL | PS | PI | PC | AECA | dsDNA | $\beta_2$GPI | HigM |
| APS | 1.3 ± 0.2 | 1.4 ± 0.15 | 1.2 ± 0.19 | 0.3 ± 0.04 | 1.05 ± 0.18 | 0.35 ± 0.05 | 1.2 ± 0.23 | 1.1 ± 0.09 |
| Control | 0.13 ± 0.02 | 0.2 ± 0.025 | 0.1 ± 0.02 | 0.15 ± 0.01 | 0.15 ± 0.03 | 0.12 ± 0.02 | 0.25 ± 0.03 | 1.15 ± 0.02 |

CL: cardiolipin, PS: phosphatidylserine, PI: phosphatidylinositol, PC: phosphatidylcholine, AECA: anti-endothelial cell antibodies, dsDNA: double stranded DNA, $\beta_2$GPI: $\beta_2$-glycoprotein-I, HIgM: human IgM antibodies.

EC is present in the sera of APS mice, compared to controls. These are either specific AECA that bind EC (Bordron *et al.*, 1998), or are aPL antibodies that crossreact with ECs through β2GPI attached to EC (Del Papa *et al.*, 1995).

### Pathological Findings

### *Histological and Immunofluorescence Findings*

#### H&E Staining

To observe gross pathological changes in cortical specimens, H&E staining was performed on specimens derived from the cortex of APS and control mice. Mild inflammatory reactions were observed in 4 out of 10 cortical specimens of APS mice. Mononuclear infiltrates were demonstrated in the meninges and subependymal areas. In cortical specimens derived from control mice no such infiltrates were discerned (Fig. 2A, B). By this staining, no thrombus was observed in the brain tissue specimens.

#### Immunofluorescence Staining

To show immune reactants deposited in the cerebral tissue, immunofluorescence staining was performed using FITC-conjugated anti-mouse IgG. In APS mice, prominent deposits of mouse IgG were shown in large and small cerebral blood vessel walls along the endothelial lining (Fig. 3A, B). Mild perivascular leakage of IgG was also observed in the vicinity of affected blood vessels (Fig. 3A, B). No such staining was observed in the control mice brain.

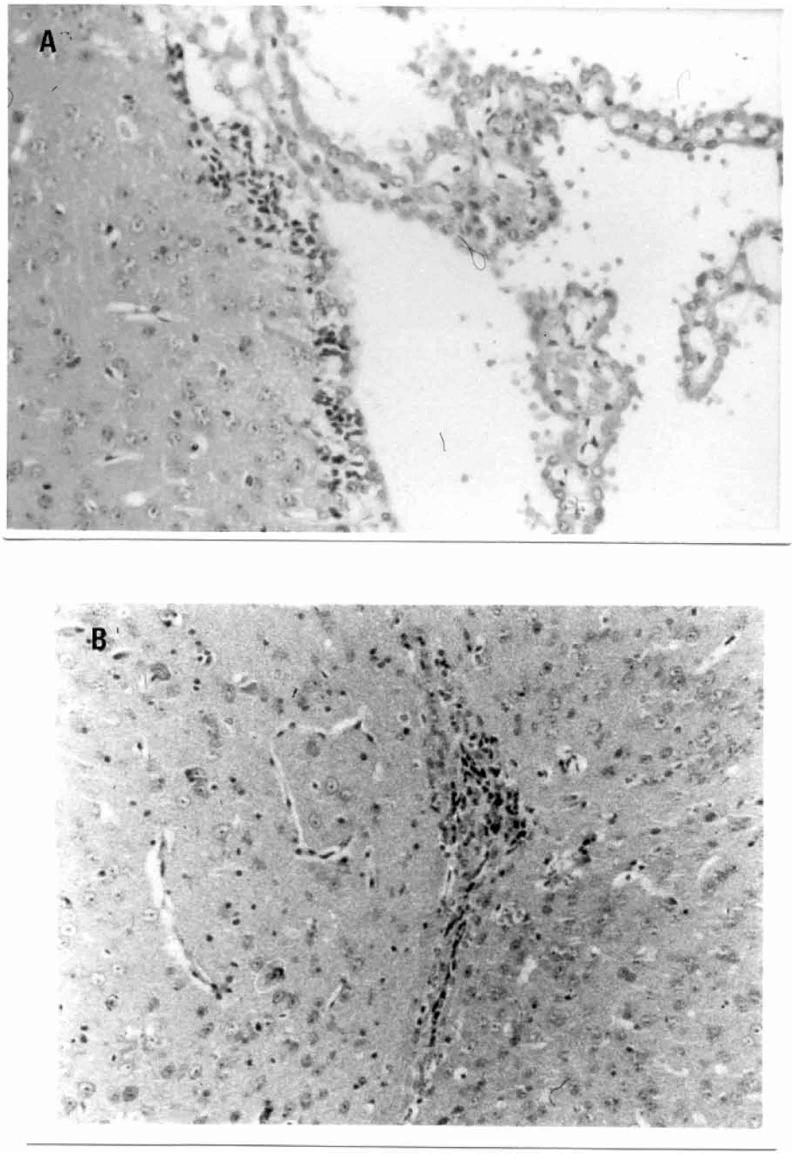

FIGURE 2 H&E staining of a representative brain specimen derived from APS mouse immunized with H-3- monoclonal aCL. (A) Subependymal infiltration of mononuclear cells is observed (arrow). (B) Infiltration of mononuclear cells is also seen in the meninges. (A,B) Original magnification, × 200.

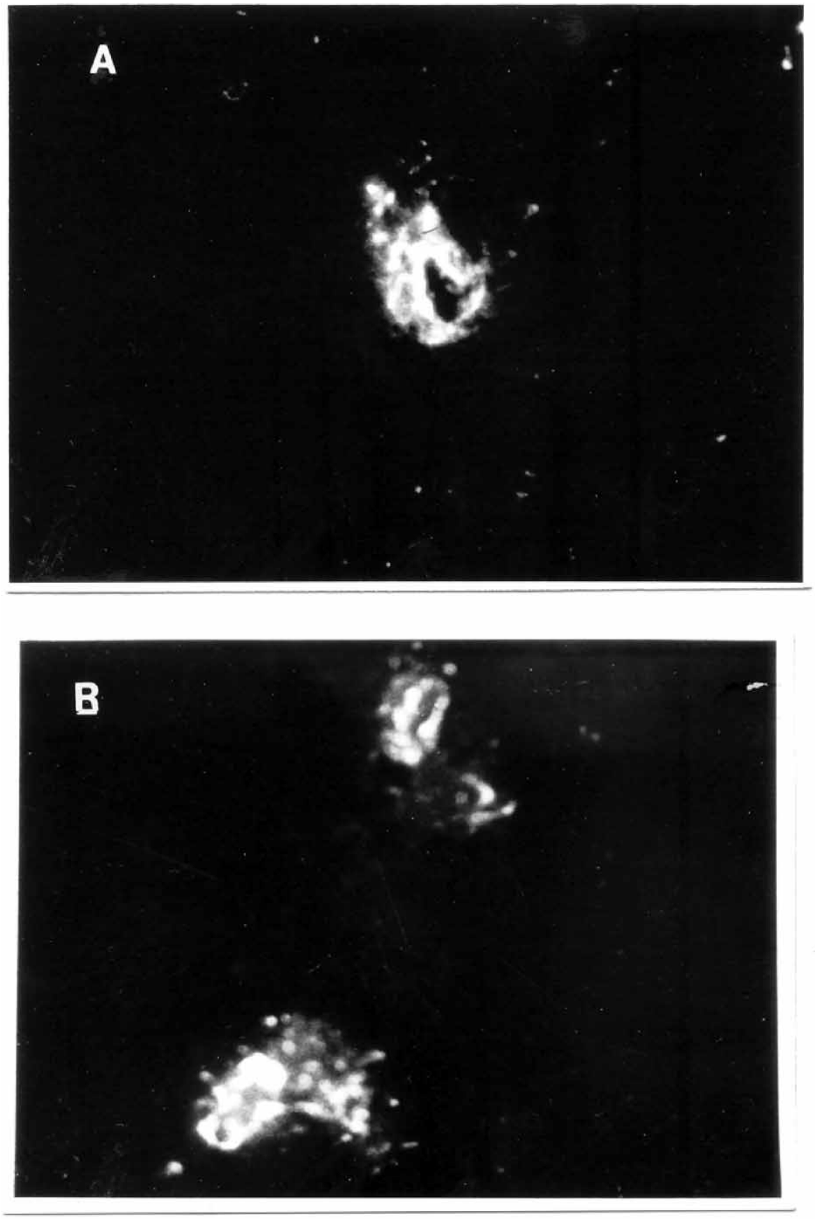

FIGURE 3 Immunofluorescence staining of a representative brain tissue specimen from mouse immunized with aCL (H-3). Prominent staining of IgG deposits is shown in the walls and endothelial lining of large vessels (A), as well as of smaller blood vessels (B). No such staining was seen in brain specimens of the controls.

### *TEM Findings*

To analyze further the changes in the cortical tissue of APS mice, TEM examination was conducted. Vasculopathy of the cortical vessels was found in specimens derived from 4 out of 10 APS mice immunized with H-3 aCL (Ziporen *et al.*, 1997). In cortical specimens of APS mice, up to 35% of capillaries shown in the tested field were found contracted, occluded by microthrombi and/or swollen EC, compared to 5% contracted capillaries in the control specimens, correlating with significant neurological and behavioral dysfunction, as previously reported (Ziporen *et al.*, 1997). As shown in Fig. 4, in the brain of APS mice the capillaries were found contracted and occluded by swollen EC leading to the formation of slit-like lumens (Fig. 4A, B), compared to wide-open lumens in most of control vessels. Aggregation of activated platelets with cytoplasmic microvesicles and the formation of platelet microthrombi were also observed in the capillaries of APS specimens (Fig. 4C). Activated macrophages were shown in the vicinity of occluded vessels (Fig. 4D). Extravasated red blood cells were seen (Fig. 4E) in areas in which the parenchyma next to the occluded capillaries showed ischemic damage (Fig. 4F).

### DISCUSSION

An experimental model for APS has been previously established in our laboratory (Bakimer *et al.*, 1992;

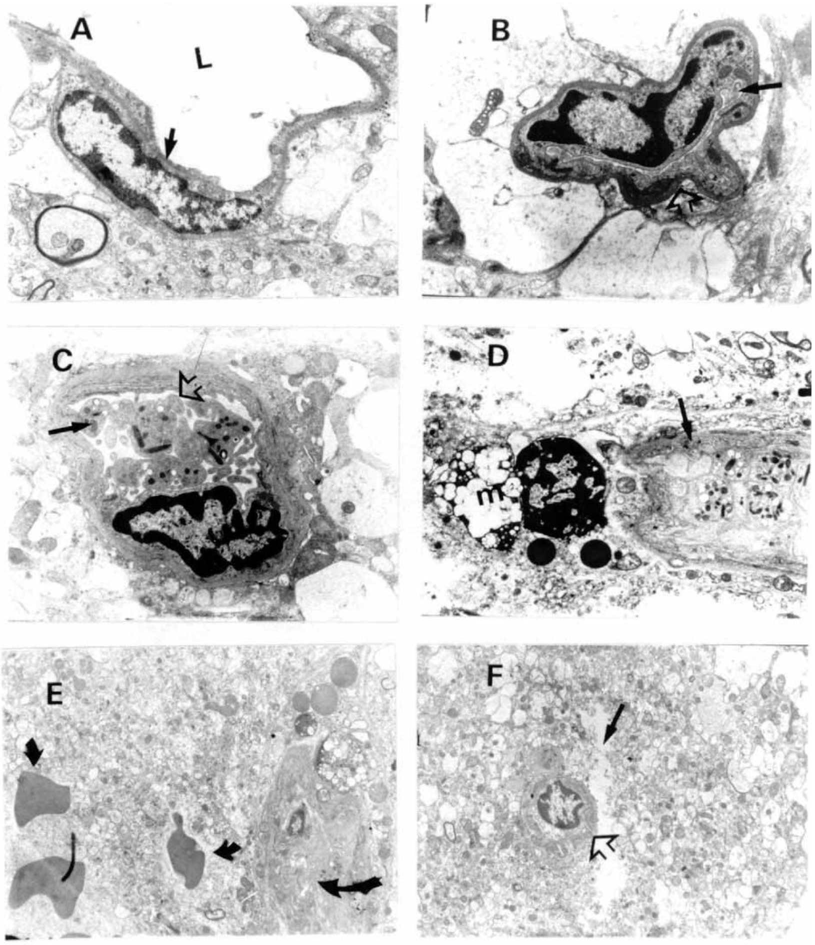

FIGURE 4 Transmission electron micrographs of representative specimens derived from the frontal brain cortex of BALB/c female mice immunized with monoclonal pathogenic aCL(H-3), or controls. (A) A capillary in a representative brain specimen derived from a control mouse is shown. Note the wide open lumen (L) surrounded by an endothelial cell (arrow) $\times$ 6000. (B) A brain specimen derived from the cortex of APS mice showing a capillary with an occluded lumen (arrow) surrounded by swollen endothelial cells, leading to the formation of a slit-like lumen. Note the zinkling of basement membrane. (arrowhead). $\times$ 12000. (C) A capillary containing an aggregate of platelets. Note inside the platelets granules (arrows), vesicles and pseudopodia-like processes (arrowhead) $\times$ 7500. (D) Agglutination of platelets forming a thrombus (arrow). The platelet membranes are preserved and adhere to the endothelial cell. An activated macrophage (*M*) is discerned $\times$ 5000. (E) extravasation of red blood cells (arrowheads) near occluded vessel (arrow) and (F) damaged tissue (arrow) near an occluded capillary (arrowhead) ( $\times$ 4000).

Ziporen *et al.*, 1997), by immunizing BALB/C mice with monoclonal aCL, carrying a pathogenic idiotype (H-3). The mice developed several clinical manifestations characteristic of the human disorder, such as thrombocytopenia, fetal loss and elevated levels of autoantibodies, including aPL, AECA and anti-$\beta_2$GPI antibodies (Bakimer *et al.*, 1992; Ziporen *et al.*, 1997). Recently, we have shown that neurological dysfunction and hyperactive behavior are also associated with experimental APS, supporting the concept that neurological involvement is one of the manifestations of APS1, 2, 8–10).

The mechanisms underlying neurological manifestations in patients with APS are not yet fully understood. Unraveling the type of pathology associated with neurological disease in APS is crucial for choosing the appropriate form of drug treatment for the patients.

Since neuropathologic studies in patients with APS and neurological disturbances are limited (Coull *et al.*, 1987; Leach *et al.*, 1989; Lie, 1989; Levine *et al.*, 1990; Westerman *et al.*, 1992; Hugston *et al.*, 1993; Lie, 1994), we aimed to investigate this unresolved question utilizing our experimental model for primary APS in mice.

Some evidence exists in the literature regarding histopathological processes associated with experimental APS that is secondary to lupus in lupus-prone mice (Kier, 1990; Smith *et al.*, 1990; Brey and Teale, 1992; Hess *et al.*, 1993; Aron *et al.*, 1995), including the MRL/lpr, its congenic strain MRL++ mice immunized with β2GPI (Smith *et al.*, 1990; Brey and Teale, 1992; Hess *et al.*, 1993; Aron *et al.*, 1995), and NZB/NZW F1 mice (Kier, 1990). In these mice neurological defects and cognitive impairment correlated with the finding of perivascular lymphocytic infiltrates in the choroid plexus, or around cerebral and hippocampal blood vessels and occlusive vasculopathy (Kier, 1990; Smith *et al.*, 1990; Brey and Teale, 1992; Hess *et al.*, 1993; Aron *et al.*, 1995). However, the correlation with aCL was not elucidated in these mice models of secondary APS.

In the current study we have extended histopathological assessment to brain tissue of naive BALB/C mice, not genetically prone to develop an autoimmune disease, that develop primary APS and neurological defects upon induction with pathogenic idiotype (Bakimer *et al.*, 1992; Ziporen *et al.*, 1997).

Experimental APS was generated in female BALB/C mice that developed elevated levels of autoantibodies, including aPLs, anti-β2GPI and AECA antibodies and low levels of a dsDNA, as tested by ELISA (Table I). Prominent binding of mice serum to HUVEC endothelial cells was further confirmed by FACS analysis (Fig. 1). Clinical manifestations of APS were confirmed, as previously reported, and significant neurological defects were demonstrated including hyperactive behavior, impaired neurological reflexes, and motor incoordination (Ziporen *et al.*, 1997).

The neurological deficits displayed by APS mice were found related mainly to the cortical level (Ziporen *et al.*, 1997). Therefore, we focused our attention on pathological changes in the cortex of APS, compared to control mice by a series of histopathological and ultrastructural analyses.

H&E staining of cortical specimens derived from APS mice revealed mainly mild inflammation, shown as small infiltrates of lymphocytes localized in the meninges and in subependymal areas (Fig. 2A, B). No wall thickening in the capillaries was evident, arguing against the possibility of classical vasculitis in the brain vessels associated with experimental APS (Lie, 1989, 1994).

Immunofluorescence staining of these specimens for mouse IgG revealed prominent immunoglobulin deposits in the EC lining of blood vessels, in contrast to the control mice specimens (Fig. 3A, B). In addition, we observed possible leakage of IgG into the adjacent brain tissue (Fig. 3A), similar to the reported perivascular deposits of IgG in the brains of MRL/lpr mice (Vogelweid *et al.*, 1991). Immune deposits in blood vessels may account for EC damage occurring in these mice, leading to a procoagulant state characteristic of APS. Moreover, once the endothelial layer has been damaged, blood components might have access to the brain tissue and affect neural tissue. Indeed, in *in vitro* studies (Kent *et al.*, 1997) aPL antibodies react directly with different epitopes in the cerebral tissue and interfere with functional properties of the neurons (Sun *et al.*, 1992; Liou *et al.*, 1994; Kent *et al.*, 1997; Chapman *et al.*, 1999). This property attributed to aPL may explain the neurological symptoms associated with APS which are not explicable by a thrombotic process.

Our ultrastructural studies revealed significant vasculopathy in the cerebral capillaries of APS mice that correlated with neurological dysfunction (Ziporen *et al.*, 1997). Many microvessels were found occluded by platelet microthrombi (Fig. 4) and the neuronal tissue adjacent to these vessels was found damaged. Activated platelets were evident as aggregates in the cerebral capillaries. The same phenomenon is seen in patients with APS associated with thrombocytopenia and are a prerequisite for vascular thrombosis in the pathogenesis of haemostatic disorders in APS (Elison *et al.*, 1995; Silver *et al.*, 1995; Fanelli *et al.*, 1997)

Thrombotic changes in the microvasculature in combination with leakage of aPL to the neuronal tissue and mild inflammation in the meninges were found as the main pathological changes in APS mice. These changes may account for the hyperactivity and other neurological defects observed in these animals (Ziporen *et al.*, 1997). Diverse autoimmune pathways, in which aPL, AECA or anti-$\beta_2$GPI antibodies are probably involved, damage the neurons (Del Papa *et al.*, 1995; Simantov *et al.*, 1995; Roubey and Hoffman, 1997; Bordron *et al.*, 1998). The autoantibodies may interfere in haemostasis leading to the occlusion of microvessels, or bind directly to the neurons. These results are in accordance with finding in autopsies of APS patients, in whom the pathologic process of aPL-associated neurologic disease resembles a non-vasculitic, mild inflammatory thrombosis of small vessels, in which the endothelial lining of small vessels is affected causing multiple micro-infarcts in the cortex (Coull *et al.*, 1987; Leach *et al.*, 1989; Lie, 1989; Westerman *et al.*, 1992; Hugston *et al.*, 1993; Lie, 1994)

### Acknowledgements

This work was supported in part by the Freda and Leon Schaller Grant for Autoimmunity Research and a grant given by the Chief Scientist of the Ministry of Health No. 3687 and by the Miriam Trajanski de Gold Fund for neurological research, Argentina.

### References

Aron, A.L., Cuellar, M.L., Brey, R.L., *et al.* (1995) “Early onset of autoimmunity in MRL/++mice following immunization with β2 glycoprotein”, *Clin. Exp. Immunol.* **101**, 78–81.

Asherson, A.R. and Cervera, R. (1994) ““Primary”, “Secondary” and other variants of the antiphospholipid syndrome”, *Lupus* **3**, 293–298.

Asherson, R.A., Cervera, R., Piette, J.C. and Shoenfeld, Y., eds, (1996) The Antiphospholipid Syndrome (CRC Press, Inc.), p 331.

Bakimer, R., Fishman, P., Blank, M., Srendi, B., Djaldetti, M. and Shoenfeld, Y. (1992) “Induction of primary antiphospholipid syndrome in mice by immunizing with a human monoclonal anticardiolipin antibodies (H3)”, *J. Clin. Investig.* **89**, 1558–1563.

Bordron, A., Dueymes, M., Levy, Y., *et al.* (1998) “Anti-endothelial cell antibody binding makes negatively charged phospholipids accessible to antiphospholipid antibodies”, *Arthritis Rheum.* **41**, 1738–1747.

Brey, R.L. (2000) “Differential diagnosis of central nervous system manifestations of the antiphospholipid antibody syndrome”, *J. Autoimmun.* **15**, 133–138.

Brey, R.L. and Teale, J.M. (1992) “Nervous system pathology in MRL/lpr and MRL/++ mice”, *Clin. Exp. Rheumatol.* **10**, 641–645.

Brey, R.L., Gharavi, A.E. and Lockshin, M.D. (1993) “Neurologic complications of antiphospholipid antibodies”, *Rheum. Dis. Clin. N. Am.* **19**, 833–850.

Chapman, J., Cohen-Armon, M., Shoenfeld, Y. and Korczyn, A.D. (1999) “Antiphospholipid antibodies permeabilize and depolarize brain synaptoneurosomes”, *Lupus* **8**, 127–133.

Coull, B.M., Bourdette, D.N., Goodnight, S.H., Briley, D.P. and Hart, R. (1987) “Multiple cerebral infarctions and dementia associated with anticardiolipin antibodies”, *Stroke* **18**, 1107–1112.

Cuadrado, M.J., Lopez-Pedrera, C., Khamashta, M.A., *et al.* (1997) "Thrombosis in primary antiphospholipid syndrome", *Arthritis Rheum.* **40**, 834–841.

Damianovich, M., Guilburd, B., George, J., *et al.* (1996) "Pathogenic role of antiendothelial cell antibodies (AECA) in vasculitis: an idiotypic experimental model", *J. Immunol.* **156**, 4946–4951.

Del Papa, N., Guidali, L., Spatola, L., *et al.* (1995) "Relationship between anti-phospholipid and anti-endothelial cell antibodies III: β2-glycoprotein-I mediates the antibody binding to endothelial membranes and induces the expression of adhesion molecules", *Clin. Exp. Rheumatol.* **13**, 179–185.

Digre, K.B., Durcan, F.J., Branch, D.W., Jacobson, D.M., Varner, M.W. and Baringer, J.R. (1989) "Amaurosis fugax associated with antiphospholipid antibodies", *Ann. Neurol.* **25**, 228–232.

Elison, D., Gatter, K., Heryet, A. and Esiri, M. (1995) "Intramural platelet deposition in cerebral vasculopathy of antiphospholipid syndrome", *Semin. Arthritis Rheum.* **24**, 273–281.

Fanelli, A., Bergamini, C., Rapi, S., *et al.* (1997) "Flow cytometric detection of circulating activated platelets in primary antiphospholipid syndrome. Correlation with thrombocytopenia and anticardiolipin antibodies", *Lupus* **6**, 261–267.

Ginsburg, K.S., Liang, H.M., Newcomer, L., *et al.* (1992) "Anticardiolipin antibodies and the risk for ischemic stroke and venous thrombosis", *Ann. Int. Med.* **117**, 997–1002.

Herranz, M.T., Rivier, G., Khamashta, M.A., Blaser, K.U. and Hughes, G.R.V. (1994) "Association between antiphospholipid antibodies and epilepsy in patients with SLE", *Arthritis Rheum.* **37**, 569–571.

Hess, D.C., Taormina, M., Thompson, J., *et al.* (1993) "Cognitive and neurologic deficits in the MRL/lpr mouse: clinicopathologic study", *J. Rheumatol.* **20**, 610–617.

Hojnik, M., George, J., Ziporen, L. and Shoenfeld, Y. (1996) "Heart valve involvement (Libman-Sack Endocarditis) in the antiphospholipid syndrome", *Circulation* **93**, 1579–1587.

Hughes, G.R.V. (1993) "The antiphospholipid syndrome: ten years on", *Lancet* **342**, 341–344.

Hugston, M.D., McCarry, G.A., Sholer, C. and Brumback, M.D. (1993) "Thrombotic cerebral arteriopathy in patients with the antiphospholipid syndrome", *Mod. Pathol.* **6**, 644–653.

Inzelberg, R. and Korczyn, A.D. (1989) "Lupus anticoagulant and late onset seizures", *Acta Neurol. Scand.* **79**, 114–118.

Inzelberg, R., Bornstein, N.M., Reider, I. and Korczyn, A.D. (1992) "The lupus anticoagulant and dementia in non-SLE patients", *Dementia* **3**, 140–145.

Katzav, A., Pick, C.G., Korczyn, A.D., Oest, E., Blank, M. and Shoenfeld, Y. (2001) "Hyperactivity in a mouse model of the antiphospholipid syndrome", *Lupus* **7**, 494–499.

Kent, M., Alvarez, F., Vogt, E., Fyffe, R., Ng, A.K. and Rote, N. (1997) "Monoclonal antiphosphatidylserine antibodies react directly with feline and murine central nervous system", *J. Rheumatol.* **24**, 1725–1733.

Kier, A.B. (1990) "Clinical neurology and brain histopathology in NZB/NZW F1 lupus mice", *J. Comp. Path.* **102**, 165–177.

Kornberg, A., Blank, M., Kaufman, S. and Shoenfeld, Y. (1994) "Induction of tissue factor-like activity in monocytes by anti-cardiolipin antibodies", *J. Immunol.* **153**, 1328–1332.

Leach, I.H., Lennox, G. and Jaspan, T. (1989) "Antiphospholipid syndrome presenting with complex partial seizures and transient ischemic attacks due to widespread small cerebral arterial thrombosis", *Neuropathol. Appl. Neurobiol.* **15**, 579–584.

Levine, S.R., Deegan, M.J., Futreu, N. and Welch, K.M.A. (1990) "Cerebrovascular and neurologic disease associated with antiphospholipid antibodies: 48 cases", *Neurology* **40**, 1181–1189.

Levy, Y., George, J., Ziporen, L., *et al.* (1997) "Massive proteinuria as a main manifestation of primary antiphospholipid syndrome", *Pathobiology* **66**, 49–52.

Lie, J.T. (1989) "Vasculopathy in the antiphospholipid syndrome: thrombosis or vasculitis, or both?", *J. Rheumatol.* **16**, 713–715.

Lie, J.T. (1994) "Vasculitis in the antiphospholipid syndrome: culprit or consort?", *J. Rheumatol.* **21**, 397.

Liou, H.H., Wang, C.R., Chou, H.C., *et al.* (1994) "Anticardiolipin antisera from lupus patients with seizure reduce a GABA receptor-mediated chloride current in snail neurons", *Life Sci.* **54**, 1119–1125.

Mosek, A., Yust, I., Treves, T.A., Vardinon, N., Korczyn, A.D. and Chapman, J. (2000) "Dementia and antiphospholipid antibodies", *Dement. Geriatr. Cogn. Disord.* **11**, 36–38.

Pierangeli, S., Barker, J.H., Stikovac, D., *et al.* (1995) "Effect of human IgG antiphospholipid antibodies on a *in vivo* thrombosis model in mice", *Thromb. Haemost.* **71**, 670–674.

Roubey, R.A.S. and Hoffman, M. (1997) "From antiphospholipid syndrome to antibody-mediated thrombosis", *Lancet* **350**, 1491–1493.

Shrot, S., Katzav, A., Korczyn, A.D., *et al.* (2002) "Behavioral and cognitive deficits occur only after prolonged exposure of mice to antiphospholipid antibodies", *Lupus* **11**, 736–743.

Silver, R.K., Mullen, T.A., Caplan, M.S., O'Connell, P.D. and Ragin, A. (1995) "Inducible platelets adherence to human umbilical vein endothelium by anticardiolipin antibody-positive sera", *Am. J. Obstet. Gynecol.* **173**, 702–707.

Simantov, R., La Sala, J.M., Lo, S.K., *et al.* (1995) "Activation of cultured vascular endothelial cells by antiphospholipid antibodies", *J. Clin. Investig.* **96**, 211–221.

Smith, H.R., Hansen, C.L., Rose, R. and Canoso, R.T. (1990) "Autoimmune MRL-lpr/lpr mice are an animal model for the secondary antiphospholipid syndrome", *J. Rheumatol.* **17**, 911–915.

Sun, K.H., Liu, W.T., Tsai, C.Y., Liao, T.S., Lin, W.M. and Yu, C.L. (1992) "Inhibition of astrocyte proliferation and binding to brain tissue of anticardiolipin antibodies purified from lupus serum", *Ann. Rheum. Dis.* **51**, 707–712.

Sutjita, M., Hohmann, A.R., Comacchio Boey, M.L. and Bradley, J. (1989) "A common anti-cardiolipin antibody idiotype in autoimmune disease: identification using a mouse monoclonal antibody directed against a naturally-occuring anti-phospholipid antibody", *Clin. Exp. Immunol.* **75**, 211–216.

Toubi, E., Khamashta, M.A., Panarra, A. and Hughes, G.R.V. (1995) "Association of antiphospholipid antibodies with central nervous system disease in systemic lupus erythematosus", *Am. J. Med.* **99**, 397–401.

Verrot, D., san-Marco, M., Dravet, C., *et al.* (1997) "Prevalence and signification of antinuclear and anticardiolipin antibodies in patients with epilepsy", *Am. J. Med.* **103**, 33–37.

Vogelweid, C.M., Johnson, G.C., Besch-Williford, C.L., Basler, J. and Walker, S.E. (1991) "Inflammatory central nervous system disease in lupus-prone MRL/lpr mice: comparative histologic and immunohistochemical findings", *J. Neuroimmunol.* **35**, 89–99.

Westerman, E.M., Miles, J.M., Backonja, M. and Sundstrom, W.R. (1992) "Neuropathologic findings in multi-infart dementia associated with anticardiolipin antibody", *Arthritis Rheum.* **35**, 1038–1041.

Wilson, A., Gharavi, A.E. and Koike, T. (1999) "International consensus statement on preliminary classification criteria for definite antiphospholipid syndrom", *Arthritis Rheum.* **42**, 1309–1311.

Ziporen, L., Goldberg, I., Arad, M., *et al.* (1996) "Libman-Sacks endocarditis in the antiphospholipid syndrome: immunopathological findings in deformed heart valves", *Lupus* **5**, 196–205.

Ziporen, L., Shoenfeld, Y., Levy, Y. and Korczyn, A.D. (1997) "Neurological dysfunction and hyperactive behavior associated with antiphospholipid antibodies: a mouse model", *J. Clin. Investig.* **100**(3), 613–619.

